# Cardiac desmosomal adhesion relies on ideal-, slip- and catch bonds

**DOI:** 10.1038/s41598-024-52725-w

**Published:** 2024-01-31

**Authors:** Manuel Göz, Sylvia M. Steinecker, Greta M. Pohl, Volker Walhorn, Hendrik Milting, Dario Anselmetti

**Affiliations:** 1 Department of Physics, Experimental Biophysics and Applied Nanoscience, Bielefeld University, Universitätstraße 25, 33615 Bielefeld, Germany; 2 Erich & Hanna Klessmann Institute for Cardiovascular Research and Development, Heart and Diabetes Center NRW, University Hospital of the Ruhr-University Bochum, Georgstraße 11, Bad Oeynhausen, Germany

**Keywords:** Molecular biophysics, Single-molecule biophysics, Cardiovascular genetics

## Abstract

The cardiac muscle consists of individual cardiomyocytes that are mechanically linked by desmosomes. Desmosomal adhesion is mediated by densely packed and organized cadherins which, in presence of Ca^2+^, stretch out their extracellular domains (EC) and dimerize with opposing binding partners by exchanging an N-terminal tryptophan. The strand-swap binding motif of cardiac cadherins like desmocollin 2 (Dsc2) (and desmoglein2 alike) is highly specific but of low affinity with average bond lifetimes in the range of approximately 0.3 s. Notably, despite this comparatively weak interaction, desmosomes mediate a stable, tensile-resistant bond. In addition, force mediated dissociation of strand-swap dimers exhibit a reduced bond lifetime as external forces increase (slip bond). Using atomic force microscopy based single molecule force spectroscopy (AFM-SMFS), we demonstrate that Dsc2 has two further binding modes that, in addition to strand-swap dimers, most likely play a significant role in the integrity of the cardiac muscle. At short interaction times, the Dsc2 monomers associate only loosely, as can be seen from short-lived force-independent bonds. These ideal bonds are a precursor state and probably stabilize the formation of the self-inhibiting strand-swap dimer. The addition of tryptophan in the measurement buffer acts as a competitive inhibitor, preventing the N-terminal strand exchange. Here, Dsc2 dimerizes as X-dimer which clearly shows a tri-phasic slip-catch-slip type of dissociation. Within the force-mediated transition (catch) regime, Dsc2 dimers switch between a rather brittle low force and a strengthened high force adhesion state. As a result, we can assume that desmosomal adhesion is mediated not only by strand-swap dimers (slip) but also by their precursor states (ideal bond) and force-activated X-dimers (catch bond).

## Introduction

**Figure 1:**
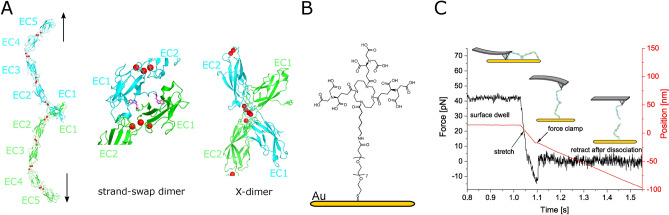
(**A**) Crystal structure of human Dsc1 ectodomain dimer which is largely homolog to Dsc2 (pdbID: 5IRY)^32^. Black arrows indicate the direction force on the Dsc2 dimer and bound Ca^2+^ ions are represented as red spheres. A His6-tag is located at the C-terminal end of the EC5 domain. Proposed binding configurations for Dsc2. Strand-swap dimers (N-terminal tryptophan moieties are highlighted in magenta) exhibit slip dissociation while X-dimers dissociate in a slip-catch-slip manner. (**B**) Schematic of cantilever and surface modification. PEG-NHS-Ester Disulfide is coupled to a gold surface and subsequently modified with Tris-NTA amine. His6-tagged desmocollin 2 bind to Ni^2+^-loaded NTA-groups^30^. The calculated contour length is approx. 15 nm. (**C**) Experimental scheme and characteristic data of a force clamp cycle (only retract). The force (black) and the cantilever position (red, zero indicates the sample surface) relative to the surface are plotted vs. time. After the surface dwell time the molecular complex is stretched to a predefined clamp force. In the clamp regime, the force is held constant until the complex dissociates. Finally, the cantilever is pulled back to the starting position of a new force cycle. The bond lifetime of an individual Dsc2 complex is estimated by the length of the clamp plateau.

Desmocollin 2 (Dsc2) is a calcium-dependent transmembrane adhesion protein in the cardiac desmosome. Its five extracellular (EC) domains extend into the intercellular gap and dimerize with their opposed counterpart by strand exchange of an N-terminal tryptophan residue^1^ (Fig. 1). Although the cardiac desmosome continuously withstands alternating forces, desmosomal cadherins exhibit a very low affinity and typical rupture forces in the range of only a few ten piconewton^2^. Nevertheless, due to their high density within the desmosomes and their cooperative binding, desmosomal cadherins establish a stable and durable cell–cell contact. Recent results from our group showed that the typical bond lifetime of cardiac cadherins is in the range of approx. 300 ms^2^. These data suggest that at the single-molecule level the cardiac desmosome is not a static compound but a highly dynamic ensemble that can alter its adhesive state by means of continuous association and dissociation processes. Other desmosomal as well as classical cadherins such as E- and VE-cadherins exhibit both structural (five extracellular domains) and functional (N-terminal tryptophan strand-swap binding motif) homologies to Dsc2^3^. In E- and VE- cadherins, the association process passes through several short-lived precursor states (e.g. X-dimers, S-dimers) before culminating in the strand swap-binding motif.^4,5^. Interestingly, the dissociation modes of E-cadherin strand-swap dimers and precursor states differ significantly. While the former exhibits dissociation of slip type, the precursor states dissociate either as ideal or catch bond, respectively^5^. Generally, non-covalently bound complexes can be divided into these three different types of bonds which are characterized by the evolution of the mean bond lifetime for increasing external forces. The concept of slip bonds was first introduced by Bell and extended by Evans and Ritchie^6,7^. They exhibit an exponential decrease of the average lifetime with increasing external force (Eq. 1). Among non-covalent bonds in biological systems, it is by far the most common type of dissociation. Catch bonds in contrast exhibit a counter-intuitive behavior as their mean lifetime increases for rising forces within a certain force interval. Although postulated by Micah Dembo in 1988, experimental evidence was not available until 2003^8,9^. Since then only a small number of different systems could be found^5,10–13^. Interestingly, these catch bond displaying molecular complexes go beyond pure molecular recognition. They are part of molecular systems that provide higher-level functionality like force-activated adhesion and catalytic activity, respectively^10–13^. To date, the nature of the catch bonds and the underlying processes are subject of vivid discussions^14^. On the one hand, with bi-phasic (catch-slip) and tri-phasic (slip-catch-slip) catch bonds there are two qualitatively different types of catch dissociation. On the other hand, there are a number of different mathematical models that take account of the possible catch bond causes such as molecular deformation^15^, protein-water interfaces^16^, fluctuating energy barriers^17^ or multi-well energy landscapes^13,18–23^.

Finally, the third and even rarer group are ideal bonds, whose mean lifetime is independent of the external force. Although they have also been predicted theoretically, only one paper provides experimental data so far^5,8,21^. It is therefore difficult to describe these systems quantitatively and to interpret the results in a meaningful way.

In this work, we specifically prepared and characterized different Dsc2 adhesion states by means of AFM-SMFS. Besides the slip bond exposing strand-swap dimer, we also addressed two different (precursor) adhesion states, one of which showed ideal behavior and the other showed catch bond dissociation. Our findings suggest that, in addition to the strand-swap dimer (slip dissociation), the ideal and catch bond adhesion states also contribute significantly to desmosomal adhesion.

## Results and discussion

**Figure 2:**
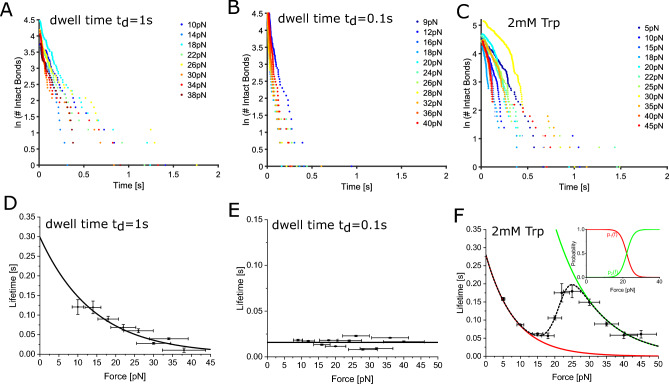
Bond survival (**A**–**C**) and force vs. lifetime (**D**–**F**) graphs of Dsc2 dimers for different experimental conditions. **(A**,**D**) Slip dissociation. (**B**,**E**) Ideal (force independent) dissociation behavior is observed for reduced interaction times. With an average lifetime of ${\tau }_{0}=0.016 \; {\text{s}}$ the ideal bonds strain resistance is reduced. (**C**,**F**) Evolution of the average bond lifetime after addition of free tryptophan. The red and green plots represent the slip dissociation from the individual binding states S1 (red) and S2 (green), respectively. The force dependent population p_1_(f) and p_2_(f) (inset) of each state allow the approximation of the tri-phasic slip-catch-slip behavior (dashed black plot).

Dsc2 dimerizes by an exchange of N-terminal tryptophan residues, which are released from their own hydrophobic binding pocket and buried within the same pocket of the opposing binding partner (Fig. 1A). This association process is self-inhibitory and possibly exhibits short-lived transition states. Here, we specifically prepare and characterize different Dsc2 binding states by means of single molecule force spectroscopy. In the first two experiments, we perform force clamp experiments in the standard HEPES buffer containing 2 mM Ca^2+^, however at different surface dwell times of ${t}_{d}=1.0\; {\text{s}}$ and ${t}_{d}=0.1\; {\text{s}}$, respectively. In a third experimental series, we added 2mM tryptophan to the HEPES buffer which serves as a competitive inhibitor of the strand-swap binding motif. We performed force clamp experiments within a force range between 5 and 45 pN. At each pulling force, we individually measured the lifetime of the Dsc2 dimers by estimating the length of the constant force plateau in the force vs. time graph (Fig. 1C). The set of lifetimes is arranged in a semi-log plot where the number of intact bonds N is plotted versus time (Fig. 2A–C, please also refer to Supplementary Information Figs. S1–S3). The average lifetime which is the inverse of the dissociation rate constant ($\tau ={k}^{-1}$) is given by the (negative) linear slope of the graph. The average lifetime versus pulling force graphs $\tau (F)$ (Fig. 2D–F) were then approximated with the appropriate model to estimate further binding parameters.

### Slip bond

For surface dwell times of ${t}_{d}=1 \; {\text{s}}$, we found exponentially decreasing bond lifetimes for increasing clamp forces over the whole range of tested forces (Fig. 2A,D). The force lifetime graph (Fig. 2D) allows to estimate the reaction length ${x}^{\ddagger }$ and the average lifetime at zero force ${\tau }_{0}$. Since this dissociation is clearly of slip bond type, we used the Kramers-Bell-Evans model gaining ${x}^{\ddagger }=0.29\pm 0.04 \; {\text{nm}}$ and ${\tau }_{0}=0.3\pm 0.06 \; {\text{s}}$, respectively (Eq. 1). These results match earlier dynamic force spectroscopy experiments from our group (Dsg2 ${\tau }_{0}=0.33\; {\text{s}}$ ) and data of other classical cadherins (Dsg1 ${\tau }_{0}=0.17$, E-cadherin ${\tau }_{0}=0.63\; {\text{s}}$ )^2,5,24,25^. Notably, the semi-log decay data often exhibit two regimes with significantly different slopes (Please refer to Supplementary Information Figs. S1–S3). The short lifetime regime displaying a steep slope turns into a rather shallow slope area for long lifetimes. However, by far the most data points are located in the steep short lifetime segment. Like others, we attribute dissociation events in the long lifetime section of the graph to nonspecific adhesion and sporadic parallel dissociation of multiple bonds which are not used for further analysis^5^. Since these (unspecific) binding events can be of different nature, a good convergence of the approximation is not necessarily to be expected. To prove the immobilization procedure and to test the specificity of the interaction we also performed a series of control experiments (please refer to Supplementary Information Fig. S5).

### Ideal bond

At reduced surface dwell times of ${t}_{d}=0.1 \; {\text{s}}$ the Dsc2 monomers only have a short interaction time before the complex is stretched to the predefined force. Within the whole tested force range the $\tau (F)$ graph does not exhibit a clear trend and can therefore be considered independent of the applied force (Fig. 2B,E). Accordingly, we estimated the bond lifetime ${\tau }_{0}=0.016\pm 0.005\; {\text{s}}$ by means of averaging the individual lifetimes at the tested forces (Fig. 2E). The interpretation of this short-lived adhesion state is rather challenging since very little has been reported about ideal bonds so far. Currently, there is only one experimental study on E-cadherin that report a similar behavior^5^. Force independent dissociation behavior is predicted for multidimensional energy landscapes when the extension of the molecular complex is equal for the transition state and the bound state^21^. In the one-dimensional case, ideal bonds can occur when the interaction energies of the bound state and the transition state are harmonic with identical spring constants and resting lengths^8^. Compared to the slip dissociation of the strand-swap binding motif, the bond lifetime of the ideal adhesion state is significantly smaller. For this reason, it seems reasonable that ideal bonds represent transient adhesion states in the energy landscape where the bond (here strand exchange) is not yet established. At reduced surface dwell times the interaction time is apparently too short to form a strand-swap dimer. The outer ECs are only loosely associated with each other. Since the strand exchange binding motif is self-inhibitory, ideal adhesion might be a precursor state that stabilizes the association process and prevents premature dissociation. Recent studies indicate that the association of classical cadherins is a multistep process that proceeds through one or more metastable states (S-dimer, X-dimer), culminating in the strand-swap dimer^4,5^.

### Catch bond

**Figure 3:**
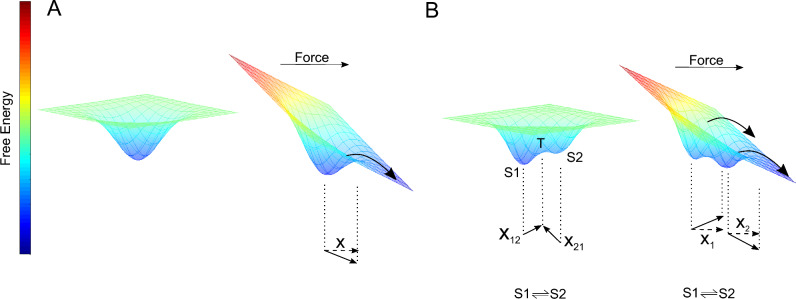
Proposed energy landscapes for slip and catch dissociation. (**A**) Single well free energy landscape of a molecular slip bond. In thermal equilibrium without an external force, the system can dissociate along a single or multiple specific dissociation paths (not shown). An external force leads to an inclination of the energy landscape that forces the bound complex across the transition state along a specific reaction path (curved arrow). The distance between the bound and the transition state (solid linear arrow) is referred to as bond length. The bond length ${x}^{\ddagger }$ derived from the experiments however is the fraction that is parallel to the applied force (dashed linear arrow). (**B**) Double well free energy landscape as proposed for catch bond interaction. The population probability of the states S1 and S2 is governed by equilibrium thermodynamics^13,33^. In order to change from one binding state to another, the system has to cross the interstate transition state T along the reaction paths x_12_ and x_21_ (linear arrows). At low external forces, almost exclusively S1 is occupied. Therefore, dissociation events from S1 dominate by far. By increasing the force, the tighter bound state S2 is successively populated. From each of the states a specific paths lead across the transition state (curved arrows). Likewise, to the single well potential the bond lengths ${x}_{1}^{\ddagger }$ and ${x}_{2}^{\ddagger }$ of the binding states S1 and S2 are defined correspondingly (solid and dashed linear arrows). Within the transition (catch) regime, both S1 and S2 are populated and dissociate along their specific paths (curved arrows). The number of dissociation events from S1 and S2 consequently depends on the occupancy of the corresponding states. As a result, the estimated complex lifetime calculates as a superposition of both binding states.

In a third experimental run, we added tryptophan (2 mM) to the HEPES buffer which acts as a competitive inhibitor for the strand exchange binding motif. Here, Dsc2 monomers associate in an alternative configuration (X-dimer) without an exchange of the N-terminal tryptophan residue. For this configuration, we found a clear tri-phasic slip-catch-slip dissociation behavior (Fig. 2C,F). A high and a low force slip bond regime are well separated by an intermediate catch interval (Fig. 2F). Since single-well energy landscape models cannot approximate tri-phasic catch bonds^14^ and due to the clear distinction between two different slip bond adhesion states, we analyzed the data within the framework of a thermodynamically derived two state two path model^13^. Here, the two slip regimes are attributed to the dissociation from two individual adhesion states S1 and S2 that are separated by an internal transition state T (Fig. 3). At both, high and low forces, only one of the two states is occupied and dissociation consequently occurs from this specific state. In the transition (catch) region, both states are populated to a certain extent and the system can dissociate from either S1 or S2. As a consequence, the estimated bond lifetime within the catch regime can be described as a probability-weighed sum of the individual lifetimes of S1 and S2. (Eqs. 2 & 3). The force dependent population probability p(f) of each adhesion state is thereby modeled by a force dependent partition function^13^. We estimated ${x}_{S1}^{\ddagger }=0.49\pm 0.03 \; {\text{nm}}$ and ${\tau }_{S1}=0.28\pm 0.02 \; {\text{s}}$, for the low force adhesion state S1 and ${x}_{S2}^{\ddagger }=0.35\pm 0.04 \; {\text{nm}}$ and ${\tau }_{S2}=2.0\pm 0.73 \; {\text{s}}$ for the high force adhesion state S2 (Fig. 2F). The transition between the two states is characterized by the gain of free energy $\Delta G=29.7\pm 1.8\; \text{kJ }{\text{mol}}^{-1}$ ($12 {k}_{B}T, T=298\; {\text{K}}$) and the compliance length $\Delta x=2.13\pm 0.01 \; {\text{nm}}$ which is the difference between the interstate reaction lengths ${x}_{12}$ and ${x}_{21}$ projected on the applied force (Fig. 3). A similar catch dissociation behavior was observed for E-cadherin, however, with some distinct differences. Firstly, the bond lifetimes are smaller over the entire force range showing maximum (catch bond peak) life times of approx. 100 ms. Furthermore, a (possible) initial slip regime at low forces is considerably less pronounced^5^. Nevertheless, when comparing the evolution of bond lifetimes of the ideal and slip bonds, respectively, E-cadherin and Dsc2 are practically identical.

Astoundingly, the loosely bound low force state S1, exhibits an almost identical bond lifetime than the strand exchange dimer. However, the respective reaction lengths ${x}^{\ddagger }$ and ${x}_{S1}^{\ddagger }$ differ considerably. As a result, both bonds show different force dependencies according to Eq. (1). The low force state (S1) of the X-dimer is therefore less stable than strand-swap dimer when subjected to an external force. Consequently, we can conclude that two different binding configurations were actually measured here. The addition of a competitive inhibitor that prevents the formation of strand exchange dimers leads to a molecular complex that clearly exhibits force-activated switching between two binding states. With a gain of free energy of approximately $30 \; \text{kJ }{\text{mol}}^{-1}$ ($12 {k}_{B}T$) when switching to the tighter bound state S2, the bond is substantially strengthened and significantly more stable against external strain.

In general, cadherins contribute significantly to the mechanical stability of tissue. It therefore seems contradictory that cadherin bonds have a short lifetime and low resistance against external forces. Before the background of the heart muscle, where the desmosome is constantly exposed to alternating forces, this force-activated self-stabilizing behavior seems not only plausible but also necessary. Furthermore, since tryptophan is a ubiquitous amino acid that is not only essential for protein synthesis, but is also part of the kynurenic metabolic pathway, which is linked to a number of diseases besides cardiac ones^26^. Even though it requires a non-physiologically high tryptophan concentration for the preparation, identification and characterization of the slip-catch-slip behavior we suppose that at physiological human tryptophan serum concentrations of approx. 60 μM at least some of the Dsc2 dimers are in the X-dimeric state^27^. Therefore, X-dimers are most likely not only a precursor state of strand-swap dimers, but due to their unique force-activated bond strengthening play a more prominent role in desmosomal adhesion. In addition, SMFS experiments on cells revealed that about 30% of the E-cadherins were present as X-dimers^28^. The general concept of force-activated self-stabilization of mechanically stressed molecular bonds by catch bonds also occurs in other biological systems such as the bacterial adhesion protein FimH or the von Willebrand factor^10,11^. From this we conclude that ideal bonds, strand-swap dimers (slip bond) and X-dimers (catch bond) most likely coexist to some degree and through their interplay provide the adhesive properties of the desmosome.

Our force spectroscopy data clearly demonstrate that Dsc2 exhibits different dissociation characteristics under different experimental conditions, indicative of different adhesion states. Apparently, the dimerization of classical cadherins is a complex, self-inhibitory process that undergoes a series of short-lived intermediate states that ultimately lead to the strand exchange dimer. By appropriate choice of experimental parameters, these intermediate states can be specifically characterized by force spectroscopy.

## Conclusion

Using AFM-SMFS, we have specifically prepared and investigated different adhesion states of the cardiac desmosomal cadherin Dsc2. We demonstrated that, in addition to the slip dissociation exhibiting strand- swap dimer, two other alternative adhesion states exist. While force independent ideal dissociation was identified for short interaction times, we were able to find tri-phasic catch bond behavior by blocking the N-terminal strand exchange binding motif. The comparatively short-lived ideal bond adhesion state is a precursor state of the strand exchange dimer which probably stabilizes the self-inhibitory association process. The catch adhesion state exhibits force-activated self-stabilization, which, in the high force regime leads to a significantly more stable connection than determined for the strand exchange dimer. Due to the ubiquitous distribution of tryptophan and recently published data for E-cadherin, we assume that the catch bonds have distinct physiological relevance in desmosomal adhesion. At this stage, however, the contribution of each adhesion mode to desmosomal adhesion can only be speculated. Binding parameters can be determined with high confidence by force spectroscopy, especially because the application of external forces mimics physiological stress. Yet, the translation of the results to cellular systems or clinical findings still remains very challenging. In order to gain a comprehensive insight into cardiac tissue integrity and to establish a consistent link between single-molecule and clinical findings, it will be necessary to perform experiments with other cadherins (*e.g.* desmoglein 2) and disease-associated variants. Furthermore, experiments with small molecular ensembles in cell culture could reveal cooperative and scale effects.

## Methods

### Recombinant expression of Dsc2

The entire extracellular domain (ECD) of Dsc2, consisting of five domains (EC1-EC5), was cloned into pLPCX (expression plasmid, Clontech, Takara Bio Kusatsu, Japan) and transfected in HT1080 cells as previously reported^29^. Expressed recombinant Dsc2 was purified by applying Immobilized Mobility Affinity Chromatography (IMAC) on HisTrap excel columns with an ÄKTA-purifier (GE Healthcare/Cytiva, Chalfont St. Giles, UK). C-terminal hexahistidin tags (RGS-(His)6) were attached to the ECDs. The purified protein solution was concentrated with Amicon Ultra-15 Centrifugal Filter Units (30000 MWCO, MerckMillipore, Darmstadt, Germany). Proteins were identified by means of sodium dodecylsulfate-polyacrylamide gelelectrophoresis (SDS-PAGE, please refer to Supplementary Information Fig. S4) followed by Western blotting and Coomassie-R250 staining (purity of solution ≥ 90%). The Dsc2-wt concentration was determined via colorimetric Bicinchoninic acid (BCA-) assay (c(Dsc2) between 0.1 gL^−1^ and 1.8 gL^−1^).

### Functionalization of AFM tips and substrates

Glas substrates (8 × 8 mm^2^) were metallized by physical vapor deposition (PVD) with a chrome layer (2 nm) followed by a gold layer (150 nm) using a conventional PVD system (MED020 Coating System, Leica Microsystems, Wetzlar, Germany). Gold coated silicon nitride cantilevers (BioLever OBL-10, Bruker GmbH, Berlin, Germany; nominal spring constant $30 \; {\text{pN}} \; {\text{nm}}^{-1}$) were rinsed with acetone and ethanol. Both, cantilever and substrates were functionalized in 15 µl of a water-free dimethyl sulfoxide (DMSO) solution containing 1mM of PEG-NHS-ester-disulfide cross-linker (4,7,10,13,16,19,22,25,32,35,38,41,44,47,50,53-Hexadecaoxa-28,29-dithiahexapentacontane-dioicacid di-N-succinimidyl ester; molecular weight: 1109.3 g mol^−1^; Polypure Inc., Oslo, Norway) and incubated it for 1h at 5–8 °C. Cantilevers and substrates were cleaned thoroughly with calcium-free HEPES buffer (10mM HEPES, 150mM NaCl, pH 7.4) in order to apply 10–15 μL of 1mM Tris-NTA-amine solution for 1h (tris-nitrilotriaceticacid-amine, Sigma-Aldrich, St. Louis, Missouri, USA). The gold substrates and cantilevers are rinsed with a nickel chloride containing HEPES buffer (10mM HEPES, 10mM NiCl_2_, 150mM NaCl, pH 7.4) in order to wash away unbound Tris-NTA. Tris-NTA-amine is a metal chelating agent, which specifically binds to His6-tagged proteins after applying Ni^2+^ ions (Fig. 1B). Even though, Tris-NTA provides a non-covalent anchorage of the proteins, it is sufficiently stable to withstand the acting forces^30^. Cantilevers and substrates could be used without loss of binding activity for up to four days. In the final preparation step, AFM probes and substrates were incubated in 10–15 μL Dsc2 solution without further dilution (0.1–1.8 gL^−1^, directly taken from purification) at 5–8 °C for 1.5 h. After extensive rinsing with HEPES measuring buffer (10mM HEPES, 2mM CaCl_2_, 150mM NaCl, pH 7.4). The specific protein coupling via the C-terminal His6-tag enables flexible spatial orientation of the binding partners and should imitate the force condition within the intercellular gap. Apart from providing a stable gold sulfur link to the cantilever and substrates, the linker adds steric elasticity to the molecular system and it ensures that dissociation events occur far from the surface. Both is required to align the force extension curves and to discriminate between specific and unspecific binding events. Furthermore, all prepared samples exhibited a dissociation event rate in the range between 5 and 10% which is essential to minimize the amount of multiple parallel dissociation events.

### Force clamp spectroscopy

SMFS experiments were conducted with the MFP-3D-BIO™ AFM system (Asylum Research, Santa Barbara, CA 93117, USA). Before each experimental series the cantilevers were calibrated by the thermal fluctuation method^31^. All force measurements were carried out in HEPES buffer (10mM HEPES, 2mM CaCl_2_, 150mM NaCl, pH 7.4). Catch bonds were observed when adding 2mM tryptophan to the buffer. Prior to buffer changes substrates and cantilevers were extensively rinsed with calcium-free washing buffer (10mM HEPES, 150mM NaCl, pH 7.4). Up to 2000 force distance cycles were recorded for pulling forces between 5 and 45 pN. Dissociation events that occurred before the preset pulling force was reached were discarded from the outset. The approach velocity and the dwell time for all experiments were set to v_appr_ = 3000 nms^−1^ and t_dwell_ = 0.1 s (ideal bond measurement), t_dwell_ = 1 s (slip bond and catch- slip bond measurements), respectively. Since molecular dissociation processes are of stochastic nature which means that they are independent of their individual force history, the retract velocity v_ret_ can in principle be set freely. Here, we chose v_ret_ = 500 nms^−1^ since this configuration ensures fast pulling and a stable force feedback control. A custom-made MATLAB software validated and analyzed the characteristic non-linear single molecule force extension curves in such a way that sporadic serial multiple rupture events and obviously malformed force curves were excluded from the analysis^13^. We estimated the complex lifetime for a given load by approximating the slope of the logarithmic decay. Simultaneous multiple rupture events that cannot be recognized from the onset appear here as outliers or shallow linear regions that can be clearly distinguished from the specific bond regime (please refer to supplementary Figs. S1–S3).

### Ideal bonds

The dissociation rate constant $k$ (which is the inverse of average bond lifetime ${\tau }_{0})$ does not exhibit any force dependence on the pulling force. Therefore, the mean bond life was estimated as the arithmetic mean of the average bond lifetimes in the given force range.

### Slip bonds

According to the Kramers-Bell-Evans model, the activation energy barrier decreases linear with an increase of the applied pulling force $f$. As a consequence, the average lifetime $\tau (f)$ decreases as:

1 $$\tau (f)={\tau }_{0} {\text{exp}}\left(-\frac{f {x}^{\ddagger }}{{k}_{B} T}\right)$$

Here, ${\tau }_{0}$ denotes the average complex lifetime at thermal equilibrium (inverse dissociation rate constant ${k}_{0}={\tau }_{0}^{-1}$), ${x}^{\ddagger }$ is the bond length in direction of the force and ${k}_{B} T$ is the thermal energy.

### Catch bonds

As mentioned earlier, there are a number of different quantitative models that describe catch bond behavior. Since the association, and probably also the dissociation of Dsc2, describes a non-trivial pathway in the energy landscape that traverses more than one intermediate state, we use the two state two path model. We approximated the tri-phasic slip-catch-slip bonds using the state two path model, where the force dependent lifetime $\tau \left(f\right)$ is calculated as a force dependent superposition of two individual slip dissociation regimes (analog to Eq. 1). The sum is weighed by the population probability of the individual adhesion states S1 and S2.

2 $$\tau \left(f\right)={p}_{1}\left(f\right){ \tau }_{1}{\text{exp}}\left(-\frac{f{x}_{1}}{{k}_{B}T}\right)+{p}_{2}(f){ \tau }_{2}{\text{exp}}\left(-\frac{f{x}_{2}}{{k}_{B}T}\right)$$

Here, ${\tau }_{\mathrm{1,2}}$ and ${x}_{\mathrm{1,2}}^{\ddagger }$ are the average lifetimes in thermal equilibrium and bond lengths of the states S1 and S2, respectively. The force dependent population probability ${p}_{\mathrm{1,2}}(f)$ of the adhesion states S1 and S2 can be calculated from the following expression:

3 $${p}_{\mathrm{1,2}}\left(f\right)={\left(1+{\text{exp}}\left(\mp \frac{\Delta {G}_{12}-f\Delta x}{{k}_{B}T}\right)\right)}^{-1}$$

Here, $\Delta {G}_{12}$ is the free energy difference between the bound states and $\Delta x$ is the compliance length which is the difference between the interstate bond lengths ${x}_{12}$ and ${x}_{21}$ projected on the applied force (Fig. 3B)^13,18^. The population probabilities ${p}_{\mathrm{1,2}}\left(f\right)$ of the adhesion states are derived from a canonical force dependent partition function. For details of the two state two path approach please refer to the work of Harder et al*.*^13^. Finally, the tri-phasic force lifetime plot is approximated by Eq. (2).

### Supplementary Information

Supplementary Figures.

## Data Availability

All datasets used for supporting the conclusions of this article are available from the Bielefeld University PUB data repository 10.4119/unibi/2983229.
