# Supplementary material for: Cardiac desmosomal adhesion relies on ideal-, slip- and catch bonds
Source: Sci Rep. 2024 Jan 31;14:2555. doi: 10.1038/s41598-024-52725-w (PMC10830561; doi:10.1038/s41598-024-52725-w)
Supplement: Supplementary file 1 — Supplementary Figures. [file 41598_2024_52725_MOESM1_ESM.docx]

**Supplementary Information**

**Cardiac desmosomal adhesion relies on ideal-, slip- and catch bonds**

Manuel Göz^1^, Sylvia M. Steinecker^1^, Greta M. Pohl^2^, Volker Walhorn^1*^, Hendrik Milting^2^ and Dario Anselmetti^1^

^1^ Department of Physics, Experimental Biophysics and Applied Nanoscience, Bielefeld University, Universitätstraße 25, Bielefeld, Germany

^2^ Erich & Hanna Klessmann Institute for Cardiovascular Research and Development, Heart and Diabetes Center NRW, University Hospital of the Ruhr-University Bochum, Georgstraße 11, Bad Oeynhausen, Germany

**Keywords:** slip bond, catch bond, ideal bond, desmocollin, cell-cell interaction, desmosome, AFM, single molecule force spectroscopy

**^*^Correspondence to:**

Volker Walhorn

Department of Physics

Experimental Biophysics and Applied Nanoscience

Bielefeld University

Universitätsstraße 25

33615 Bielefeld, Germany

Email: volker.walhorn@physik.uni-bielefeld.de

**Figure S1: Slip Dissociation**

**
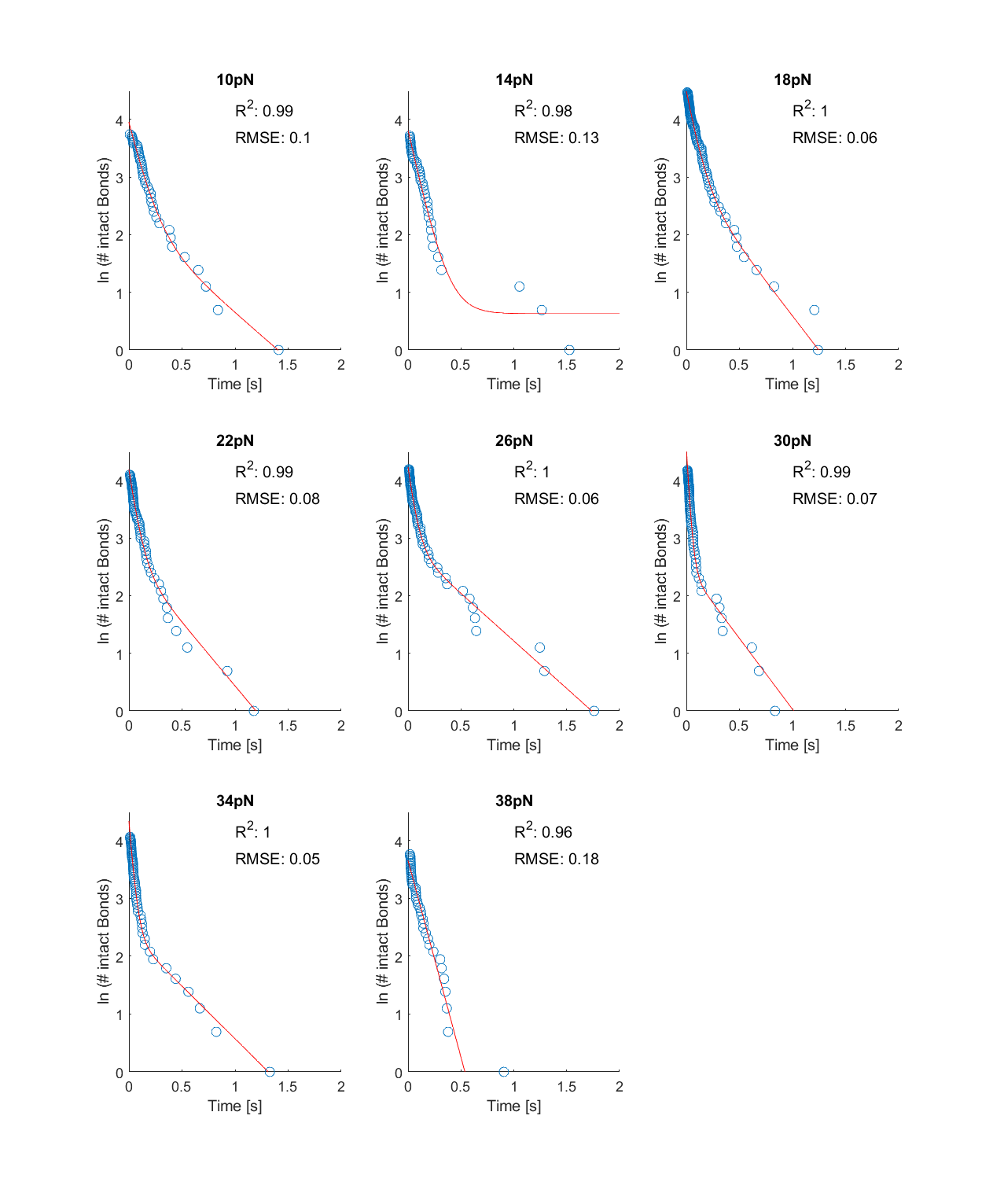
**

**Figure S1:** Full set of ln(N) vs. time graphs for slip dissociation. The natural logarithm of intact bonds is plotted vs. time (open circles). The decay curves are approximated by a single or double exponential (red line) based on the Akaike Information Criterion (AIC). The negative inverse slope of the steep regime accounts for the lifetime of the Dsc2 dimers. The shallow, long lifetime regime is due to unspecific adhesion. R^2^ and RMSE specify the coefficient of determination and the root mean square error of the approximation.

**Figure S2: Ideal Dissociation**


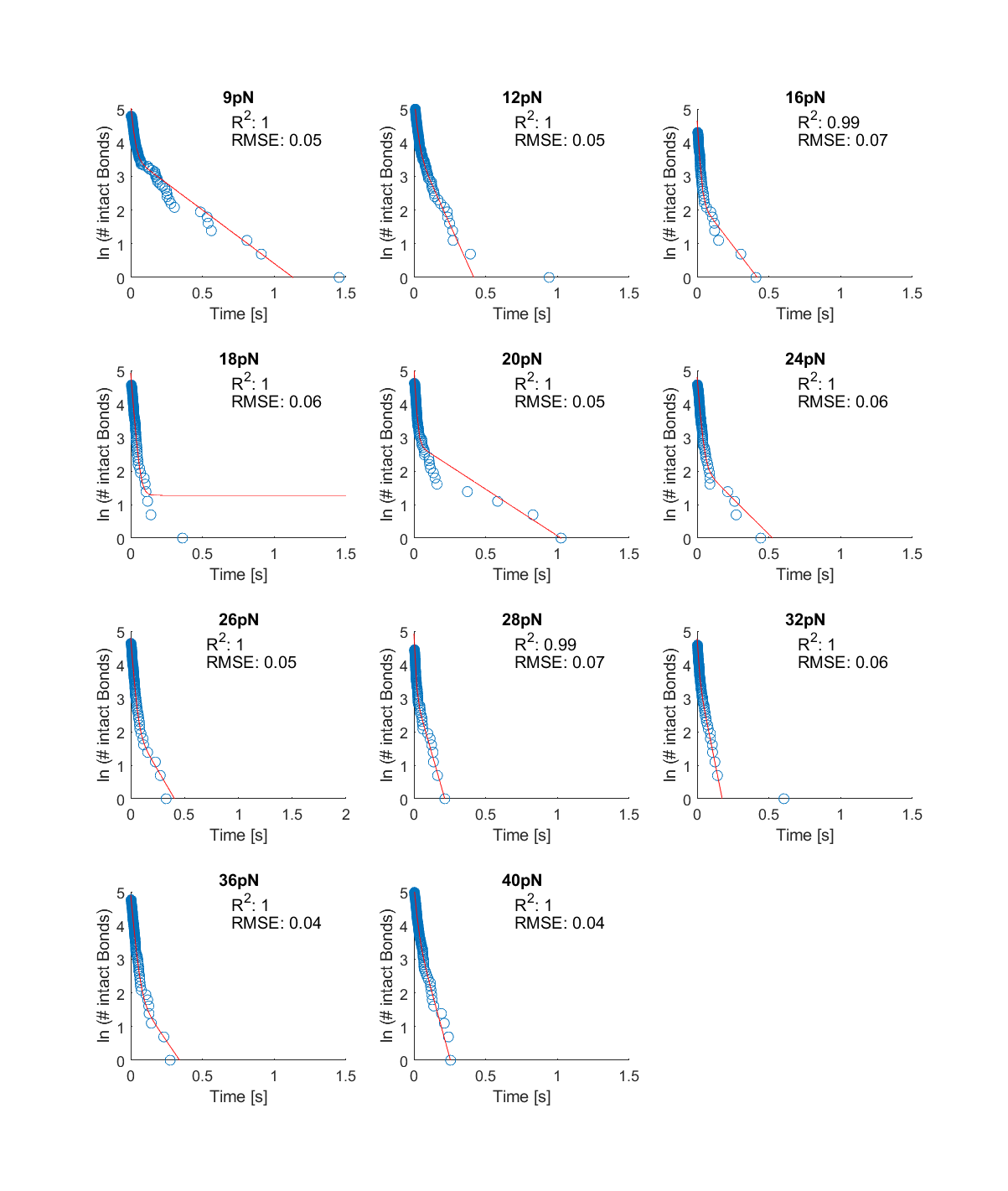


**Figure S2:** Full set of ln(N) vs. time graphs for ideal dissociation. Approximation, determination of R^2^ and the RMSE were carried out as described above.

**Figure S3: Catch Dissociation**


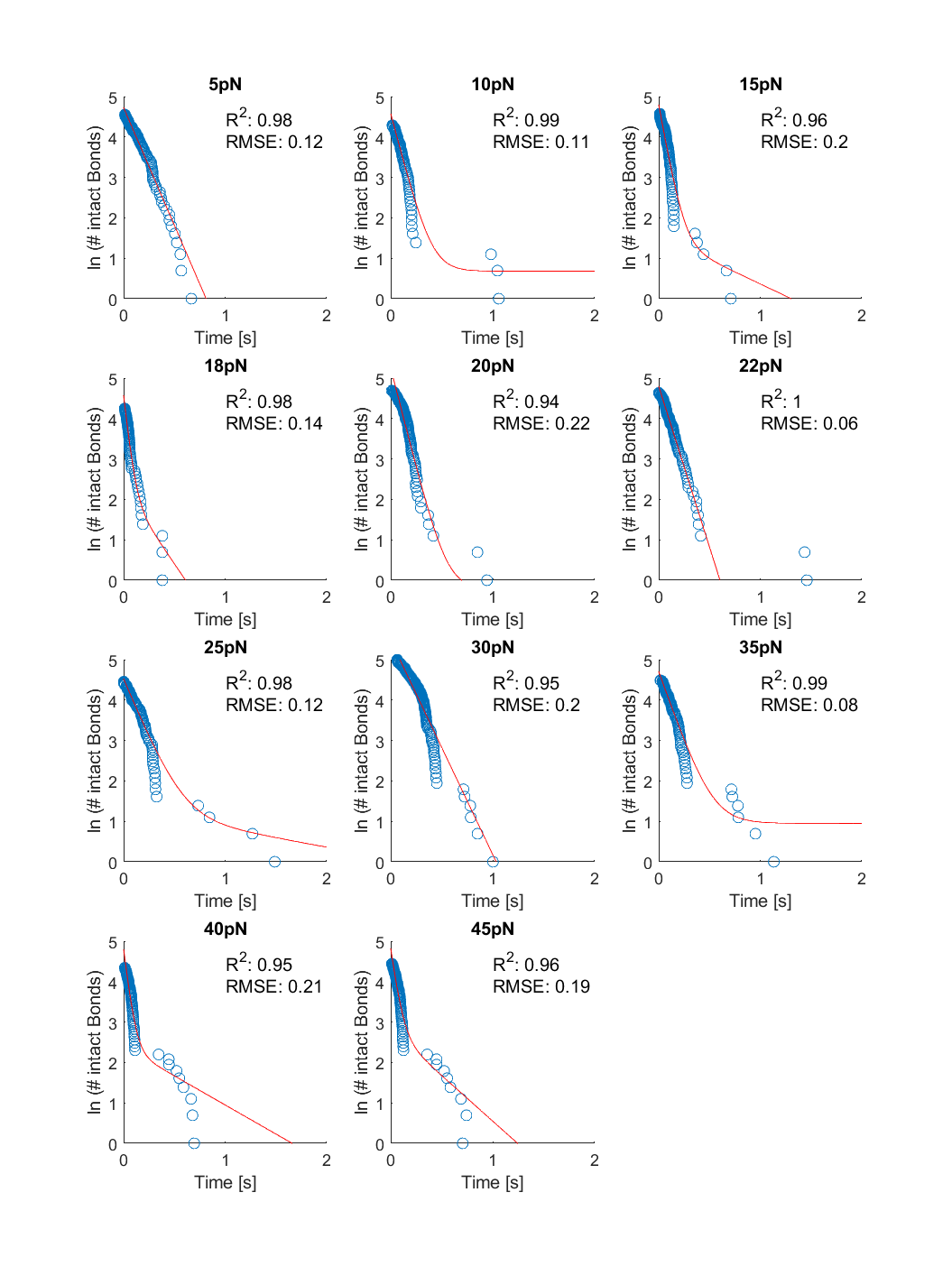


**Figure S3:** Full set of ln(N) vs. time graphs for catch bond dissociation. Approximation, determination of R^2^ and the RMSE were carried out as described for Figure S1.

**Figure S4 Protein Purification**


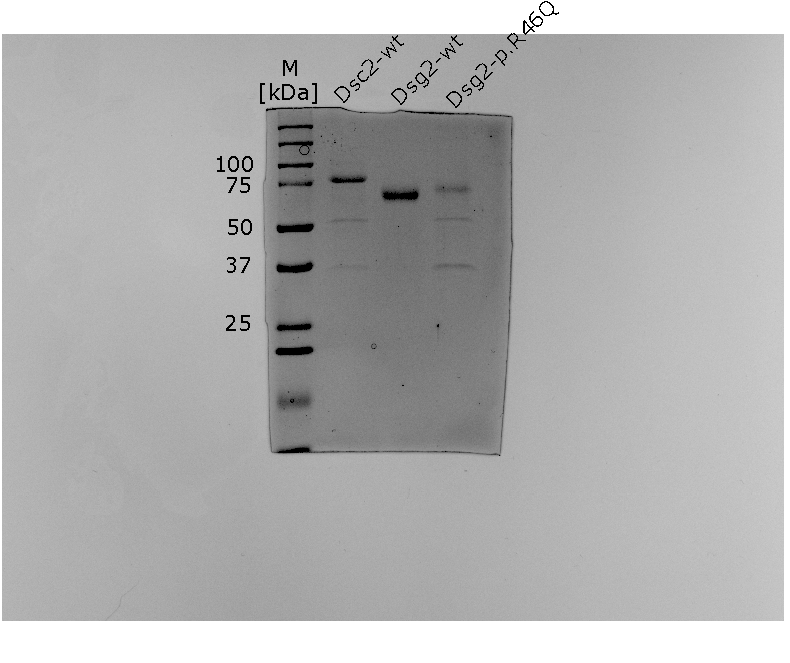


**Figure S4:** Representative SDS-PAGE of several purified recombinant cardiac cadherins (Dsc2-wt left). Protein staining by Coomassie-R250 reveals a purity ≥ 85%. Freshly isolated Dsc2-wt constructs were used for AFM analyses.

**Figure S5: Force Spectroscopy Control Experiments**

**
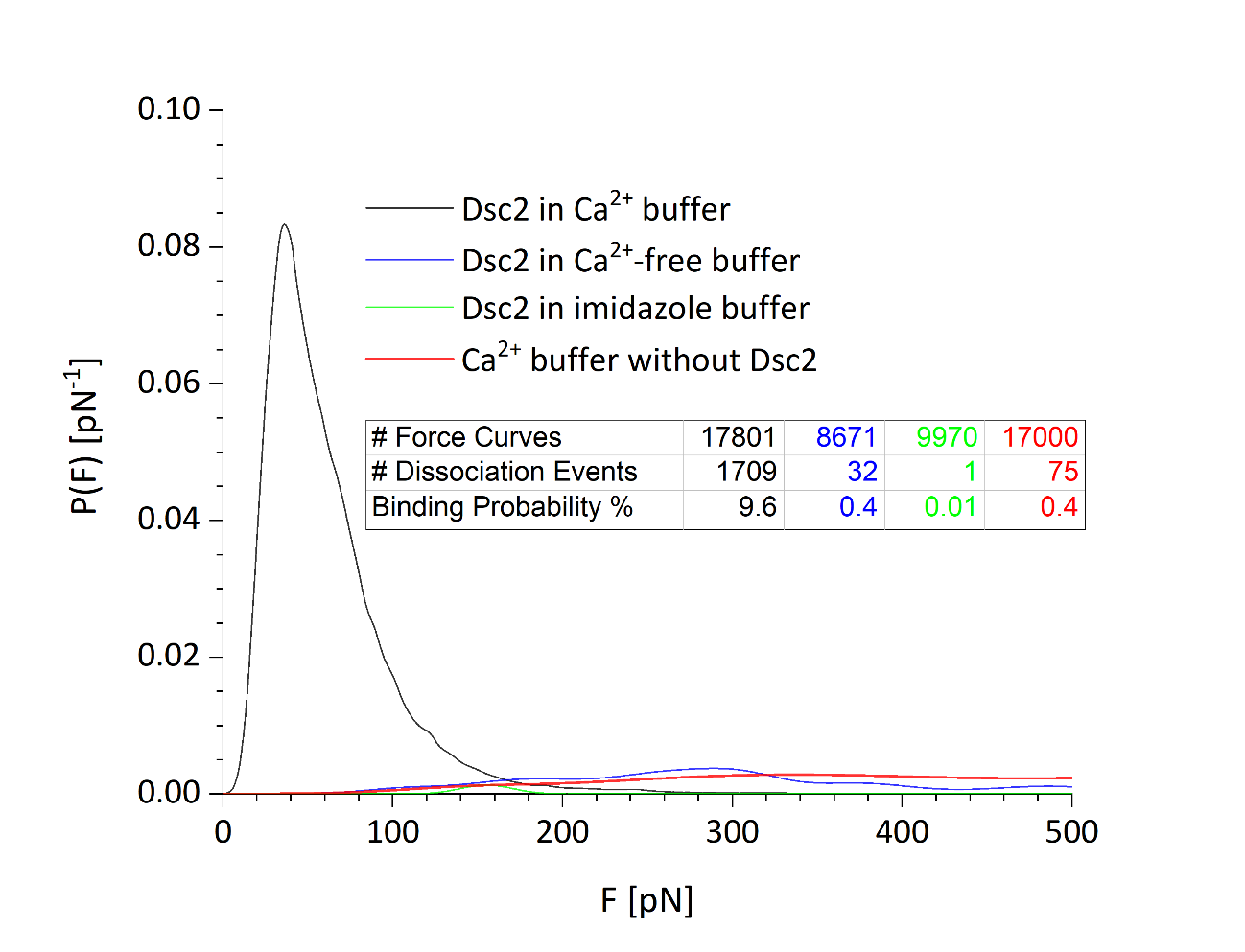
**

**Figure S5:** Series of control experiments for different Ca^2+^ concentrations and sample substrates. The experiments were conducted in dynamic force spectroscopy mode at a pulling speed of 1000 nm/s estimating the distribution of dissociation forces. The dissociation probability distributions are scaled for the absolute binding frequency. Experiments with Dsc2 modified AFM cantilever and sample substrate in 2 mM calcium-buffer (black) and calcium-free buffer (blue) expose a clear dependency of the binding frequency on the calcium concentration. The background of unspecific adhesion was tested with a readily modified cantilever on a substrate lacking Dsc2 (red) and in imidazole containing buffer detaching Dsc2 from their Tris-NTA anchor (green). The number of force curves, dissociation events and binding probabilities are listed in the table in the colours of the corresponding plots.
